# Composite Multiscale Cross-Sample Entropy Analysis for Long-Term Structural Health Monitoring of Residential Buildings

**DOI:** 10.3390/e23010060

**Published:** 2020-12-31

**Authors:** Tzu-Kang Lin, Dong-You Lee

**Affiliations:** Department of Civil Engineering, National Chiao Tung University, Hsinchu 30010, Taiwan; icrtqweasd19@gmail.com

**Keywords:** structural health monitoring, multi-scale, composite cross-sample entropy, long-term evaluation

## Abstract

This study proposesd a novel, entropy-based structural health monitoring (SHM) system for measuring microvibration signals generated by actual buildings. A structural health diagnosis interface was established for demonstration purposes. To enhance the reliability and accuracy of entropy evaluation at various scales, composite multiscale cross-sample entropy (CMSCE) was adopted to increase the number of coarse-grained time series. The degree of similarity and asynchrony between ambient vibration signals measured on adjacent floors was used as an in-dicator for structural health assessment. A residential building that has been monitored since 1994 was selected for long-term monitoring. The accumulated database, including both the earthquake and ambient vibrations in each seismic event, provided the possibility to evaluate the practicability of the CMSCE-based method. Entropy curves obtained for each of the years, as well as the stable trend of the corresponding damage index (DI) graphs, demonstrated the relia-bility of the proposed SHM system. Moreover, two large earthquake events that occurred near the monitoring site were analyzed. The results revealed that the entropy values may have been slightly increased after the earthquakes. Positive DI values were obtained for higher floors, which could provide an early warning of structural instability. The proposed SHM system is highly stable and practical.

## 1. Introduction

Infrastructures, including bridges, dams, and buildings have been widely set up to protect and facilitate our daily life. As the structural performance of a building which is frequently subjected to natural hazards or human use is likely to lose its original strength over time, how to evaluate whether the building is safe or not has become an important issue. As a result, structural health monitoring (SHM) has been extensively developed over the last few decades.

In 1993, Kim analyzed a ten-bay hexagonal truss and applied a new method to match the undamaged analytical model and the damaged test modes [1]. Zhang proposed transmittance function monitoring, which measured vibrations to detect the damage to a structure without measuring the excitation force from the sensor’s location [2]. In 2002, Lee used environmental vibration data caused by the dynamic loads of vehicles to identify the location and severity of the damage, then consider the mass effect of the dynamic load on the bridge by using the resonance frequency and the modal shape of the damage [3]. In 2003, Chang summarized the limitations and applications of vibration-based SHM methods and used the actual measured structural dynamic response to update the stiff-ness matrix, mass matrix, and damping matrix [4]. In 2010, inspired by the field of bio-medical engineering, an SHM system was implemented by integrating the naïve Bayesian (NB) classification and DNA-like expression data, taking advantage of a double-tier re-gression process to extract the expression array from the recorded structural time history [5]. In 2020, Shokravi [6] made use of extended observability matrices and used subspace system identification (SSI) to resolve the problem of SHM in the real world.

Rudolph Clausius first proposed entropy to represent the variation of an object. Entropy is an amount of energy in thermodynamics that cannot work in terms of kinetics [7]. In 1948, Shannon used the entropy that was often mentioned in thermodynamics in information theory. Entropy is defined as the average amount of information which was contained in each signal received, also called information entropy [8]. In 1991, Pincus improved the Kolmogorov entropy with approximate entropy (ApEn), which could measure the complexity and regularity of time series in real life without the need for coarse-grained processes [9]. Richman published sample entropy (SampEn) in 2000, improving the shortcomings of ApEn. The advantage of sample entropy is that the length of time series has no effect on the analysis results and thus the error of ApEn can be reduced [10,11]. In 2002, Costa et al. proposed multiscale entropy (MSE) to solve the difficulty of using single-scale sampling entropy to determine interbeat interval time series in normal people who have arrhythmia and congestive heart disease [12,13]. The measured original signal is converted to different scales and then analyzed by sampling entropy. The results showed that the accuracy when distinguishing different signals could be in-creased. The experimental verification was conducted by measuring the heart rhythm timing signal through the MSE method. The entropy value of the healthy group was the highest, and the signal which demonstrated health was the most complicated. So far, the multiscale entropy analysis method has been widely used.

In 1996, Pincus proposed cross approximate entropy (Cross-ApEn) [14]. Richman developed cross sample entropy (Cross-SampEn) to express the asynchrony and the dissimilarity between two distinct time series in 2000 [10]. The results indicated that one time series was more synchronized than the other time series, and the cross-approximate entropy and cross-sampling entropy were lower. Fabris et al. used sampling entropy and cross-sampling entropy to analyze electroglottogram and microphone signals to distinguish between a healthy control group and patients with throat or vocal cord diseases [15]. In 2013, Wu et al. applied composite multiscale entropy (CMSE) to improve the unreliability of the sampling entropy of the coarse-grained sequence when the scale factor became larger [16]. Yin et al. then proposed composite multiscale cross-sample entropy (CMSCE) to revise the standard of multiscale sampling entropy [17]. Nowadays, more and more researchers in various fields use entropy for different forms of signal analysis or malfunction monitoring. In 2017, Wimarshana et al. applied wavelet transformation and sampling entropy to detect breathing cracks on a cantilever beam and optimized the parameters of sampling entropy to increase detection efficiency [18]. Guan et al. applied cross-sample entropy to the failure detection of shear structures and input white noise to simulate random loads by using finite element models [19].

**Figure 1:**
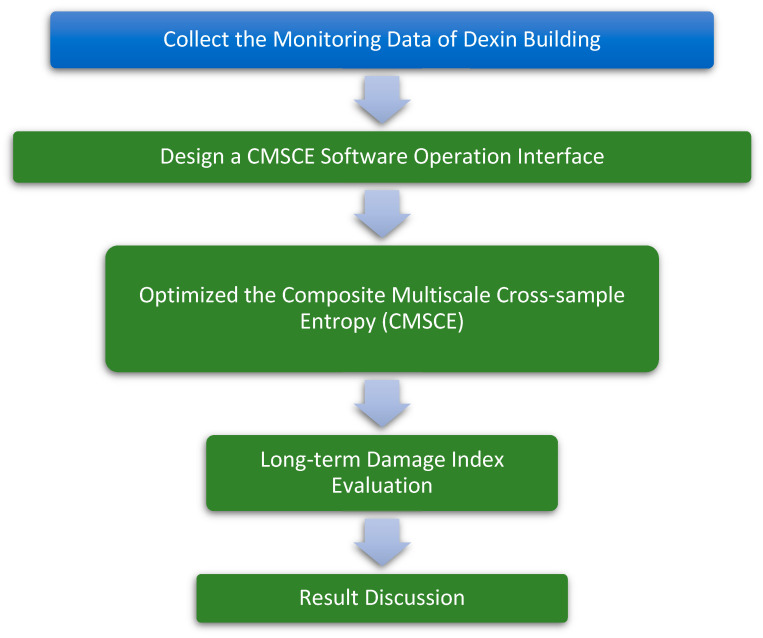
Flowchart of the study.

The aim of this study was to develop an entropy-based SHM system to effectively monitor long-term structural safety. Based on previous research and experimental results, composite multiscale cross-sample entropy (CMSCE), which had good performance in accuracy and reliability, was adopted as the core SHM algorithm. A structural health monitoring demonstration program combining CMSCE and the damage index was also developed with the support of LabVIEW software. The flowchart of the study is shown in Figure 1.

## 2. Methodology

### 2.1. Composite Multiscale Cross-Sample Entropy Method

In the concept of multiscale entropy (MSE) proposed by Costa [13], an original signal is transformed into various scales through coarse graining. Thus, the signal can be accurately analyzed. Moreover, the original time series can be divided into nonoverlapping windows with a time scale *τ* ranging from 1 to *N*. The length of each coarse-grained time series is *N*/*τ*, and a new time series $\left\{y_{j}^{\left(\tau \right)}\right\}$ can be constructed by calculating the arithmetic mean of each window.

(1) yj(τ) = 1τ∑i=(j −1)τ+1jτxi, 1 ≤ j ≤ Nτ

Wu et al. [16] proposed the concept of composite MSE (CMSE) to improve the accuracy of MSE by increasing the number of coarse-grained time series. In CMSE, a kth coarse-grained time series for a scale factor of *τ* can be defined as follows:

(2) yk,j(τ) = 1τ∑i=(j −1)τ+kjτ+k−1xi, 1 ≤ j ≤ Nτ, 1≤k≤τ

Cross-sample entropy (cross-SampEn) primarily entails evaluating the degree of asynchrony and dissimilarity between two time series from the same system. Cross-SampEn and SampEn involve similar procedures, which can be summarized as follows: First, consider two individual time series $\left\{X_{i}\right\}{\;=\;\{x}_{1}{,\;\ldots ,\;x}_{i}{,\;\ldots ,\;x}_{N}\}$ and $\left\{Y_{j}\right\}{\;=\;\{y}_{1}{,\;\ldots ,\;y}_{j}{,\;\ldots ,\;y}_{N}\}$ with a length N. These two time series can be divided into templates of length *m* for: $u_{m}\left(i\right){\;=\;\{x}_{i}{,\;x}_{i+1}{,\;\ldots ,\;x}_{i+m-1}\},\;1\;\leq \;i\;\leq \;N\;-\;m\;+\;1$ and $v_{m}\left(j\right){\;=\;\{y}_{j}{,\;y}_{j+1}{,\;\ldots ,\;y}_{j+m-1}\},\;1\;\leq \;j\;\leq \;N\;-\;m\;+\;1$. Accordingly, two template spaces $T_{x}$ and $T_{y}$ can be defined as follows:

(3) $$T_{x}\;=\;\left[\begin{array}{c} \begin{array}{cccc} x_{1} & x_{2} & \cdots & x_{m}\\ x_{2} & x_{3} & \ldots & x_{m-1}\\ \vdots & \vdots & \ddots & \vdots \\ x_{N-m+1} & x_{N-m+2} & \cdots & x_{N} \end{array} \end{array}\right]T_{y}\;=\;\left[\begin{array}{c} \begin{array}{cccc} y_{1} & y_{2} & \cdots & y_{m}\\ y_{2} & y_{3} & \ldots & y_{m-1}\\ \vdots & \vdots & \ddots & \vdots \\ y_{N-m+1} & y_{N-m+2} & \cdots & y_{N} \end{array} \end{array}\right]$$

The number of similarities between $u_{m}\left(i\right)$ and $v_{m}\left(j\right)$ is defined as $n_{i}^{m}\left(r\right)$, which can be expressed as follows:

(4) $$n_{i}^{m}\left(r\right)\;=\sum_{j=1}^{N-m}d\left[u_{m}\left(i\right){,\;v}_{m}\left(j\right)\right]$$

where $d\left[u_{m}\left(i\right){,\;v}_{m}\left(j\right)\right]$ is the maximum distance between the two templates *i* and *j.* If the maximum distance is within *r*, which is a predetermined threshold, $n_{i}^{m}\left(r\right)$ can be calculated:

(5) $$d\left[u_{m}\left(i\right){,\;v}_{m}\left(j\right)\right]\;=\;max\{|x\left(i+k\right)-y\left(j+k\right)|:\;0\;\leq \;k\;\leq \;m\;-\;1\}$$

(6) $$d\left[u_{m}\left(i\right){,\;v}_{m}\left(j\right)\right]\;\leq \;r,\;1\;\leq \;j\;\leq \;N\;-\;m$$

The probability of similarity between the templates can be calculated as follows:

(7) $$U_{i}^{m}\left(r\right)\left(v∥u\right)\;=\;\frac{n^{m}\left(r\right)}{\left(N\;-\;m\right)}$$

Subsequently, the average probability of similarity between the templates of length m can be calculated as follows:

(8) $$U^{m}\left(r\right)\left(v∥u\right)\;=\;\frac{1}{\left(N-\;m\right)}\sum_{i=1}^{N-m}U_{i}^{m}\left(r\right)\left(v∥u\right)$$

where $U^{m}\left(r\right)\left(v∥u\right)$ represents the degree of synchrony between the two pattern spaces. The same steps are executed to calculate the model space of length *m* + 1 by using the following equation:

(9) $${CS}_{E}\left(m,\;r,\;N\right)\;=\;-\ln\left\{\frac{U^{m+1}\left(r\right)\left(v∥u\right)}{U^{m}\left(r\right)\left(v∥u\right)}\right\}$$

SampEn values can be calculated for all coarse-grained time series. The mean of *τ* SampEn values represents the CMSE value, as expressed in the following equation:

(10) $$CMSE\left(x,\tau ,m,r\right)=\frac{1}{\tau }\sum_{k=1}^{\tau }SampEn\left(y_{k}^{\left(\tau \right)},m,r\right)$$

On the basis of the concept of CMSE, Yin et al. [17] proposed composite multiscale cross-SampEn (CMSCE), which can be defined as follows:

(11) $$CMSCE\left(x,y,\tau ,m,r\right)=\;\frac{1}{\tau }\sum_{k=1}^{\tau }SampEn\left(x_{k}^{\left(\tau \right)},y_{k}^{\left(\tau \right)},m,r\right)$$

The CMSCE values can be derived for the two time series. The derived values can then be plotted as a function of the scale factor $\left(f\left(\tau \right){=S}_{E}\right)$.

### 2.2. DI Measure

Studies in the field of bioinformatics have reported that the area of the MSE curve can be used as an index to quantify complexity [20,21,22]. Accordingly, the present study used a damage index (DI) proposed as a measure for identifying damaged floors on a structure [23,24]. This index is expected to reduce the probability of human errors in the monitoring of damaged floors. Two sets of CMSCE curves were plotted to represent healthy and damaged structures. For a structure with F floors, the following matrices can be used to derive the CMSCE curves for damaged and undamaged floors:

(12) $${\mathrm{CMSCE}}_{\mathrm{undamaged}}\;=\;\left\{\begin{array}{c} H_{1}\\ H_{2}\\ \vdots \\ H_{F} \end{array}\right\}{\;\mathrm{CMSCE}}_{\mathrm{damaged}\;}=\left\{\begin{array}{c} D_{1}\\ D_{2}\\ \vdots \\ D_{F} \end{array}\right\}$$

where *H* and *D* represent healthy and damaged structural conditions, respectively. Furthermore, the numerical subscripts for *H* and *D* represent the floors of the structures under analysis.

Therefore, $H_{1}$, for example, represents the cross-SampEn results for signals obtained between the foundation and the first floor of the structure under healthy conditions, and it can be expressed as follows:$H_{1}\;=\;\left\{{CS}_{E}^{H_{11}}{,\;CS}_{E}^{H_{12}}{,\;CS}_{E}^{H_{13}},\;\cdots {,\;CS}_{E}^{H_{1\tau }}\right\}$, where ${CS}_{E}^{H_{1\tau }}$ represents the cross-SampEn of time series obtained from the first floor at the scale factor *τ* under healthy structure conditions. Accordingly, the cross-SampEn of each damaged floor can be expressed as follows: $D_{F}=\;\left\{{CS}_{E}^{D_{F1}}{,\;CS}_{E}^{D_{F2}}{,\;CS}_{E}^{D_{F3}},\;\cdots {,\;CS}_{E}^{D_{F\tau }}\right\}$, where F is the floor number and ${CS}_{E}^{D_{1\tau }}$ represents the cross-SampEn of the time series obtained from the first floor at the scale factor *τ* under damaged structure conditions.

The DI can be derived using the following equation:

(13) $$DI_{F}=\sum_{q=1}^{\tau }\left(CS_{E}^{D_{Fq}}-CS_{E}^{H_{Fq}}\right)$$

The DI of each floor can be calculated by subtracting the entropy value of the damaged structure from the reference entropy value of the healthy structure on the same floor and then adding the values up. Previous research has demonstrated that the damage level and condition of a three-dimensional multibay structure can be detected by CMSCE analysis correctly. A positive DI value indicates that the floor is damaged, whereas a negative DI value indicates that the floor is undamaged [25].

## 3. Long-Term Monitoring of Practical Residential Buildings

### 3.1. Introduction of the Dexin Residential Building

**Figure 2:**
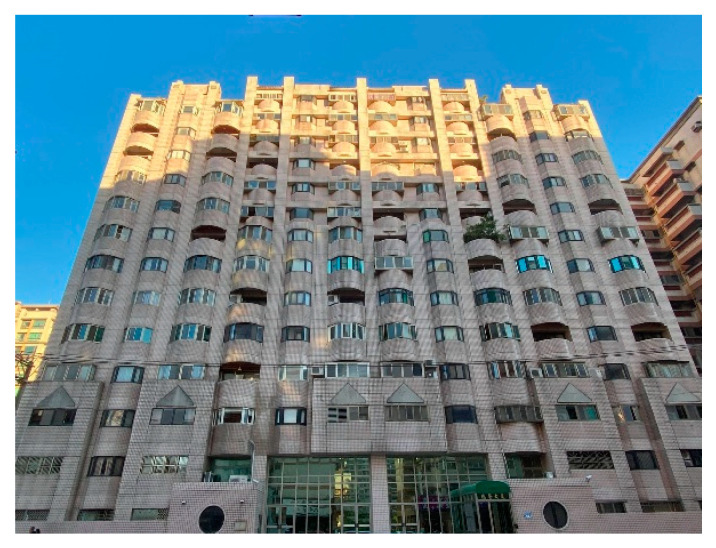
The Dexin residential building.

**Figure 3:**
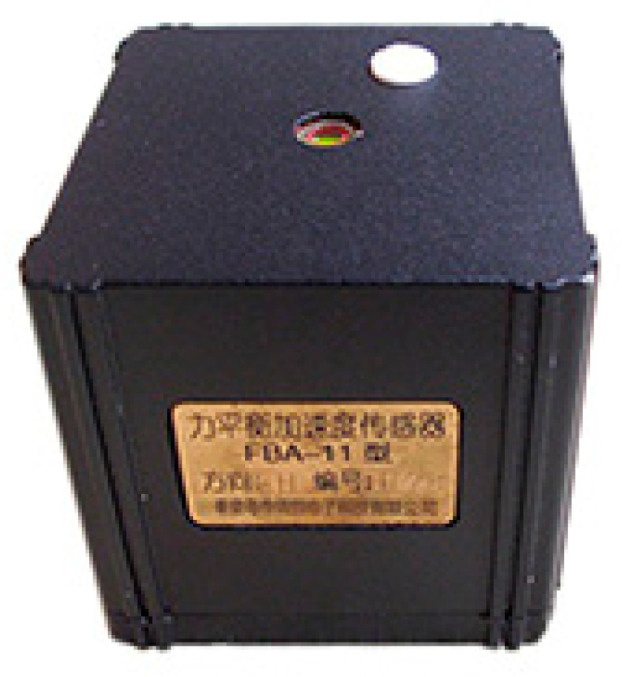
FBA-11 sensor.

**Figure 4:**
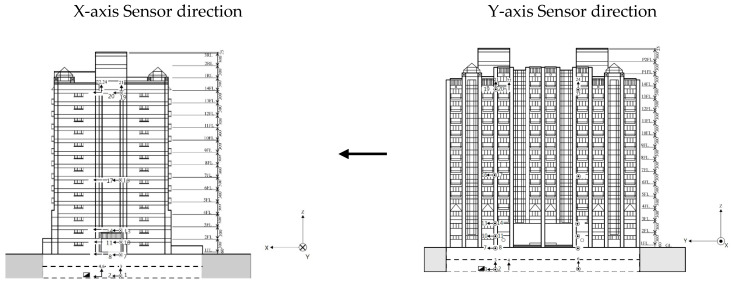
Elevation of Dexin building.

To demonstrate the practical applicability of the proposed monitoring system, this study selected a residential building—called the Dexin residential building (Figure 2)—located in Hsinchu City, Taiwan. Constructed in October 1991, the building has a reinforced concrete structure with 14 aboveground floors and 2 underground floors. The structure was designed in accordance with the ACI-318-77 and UBC technical codes. The strength of the concrete material used in the building is 280 kg/cm^2^, and the yield strength $f_{y}$ of the steel bars is 4200 kg/cm^2^. Moreover, the girder section measures 50 cm × 70 cm, the beam section measures 40 cm × 65 cm, and the ground beam section measures 100 cm × 250 cm. This structure was one of the sites of the Taiwan Strong Motion Instrumentation Program implemented by the Central Weather Bureau. During this program, 24 sensors were deployed on the structure to record its response to earthquakes. Acceleration data for each floor were measured using strong-motion seismometers (FBA-11) (Figure 3) with several channels. Specifically, acceleration data were mainly recorded by the sensors in channels 2, 8, 11, 14, 17, and 20 (X-axis) and channels 1, 7, 10, 13, 16, and 19 (Y-axis) on the second basement floor (B2), first floor (1F), second floor (2F), third floor (3F), seventh floor (7F), and fourteenth floor (14F), respectively. The orientation of each sensor and elevation of Dexin Building is shown in Figure 4. Table 1 presents the channel numbers for the measurements on each floor. The aging and potential destruction of the structure due to natural disasters, such as earthquakes and typhoons, may threaten the lives and properties of residents over time. Therefore, a long-term monitoring project was conducted to analyze CMSCE data. Complete structural health diagnosis software was integrated into the system.

### 3.2. CMSCE Labview User Interface

**Figure 5:**
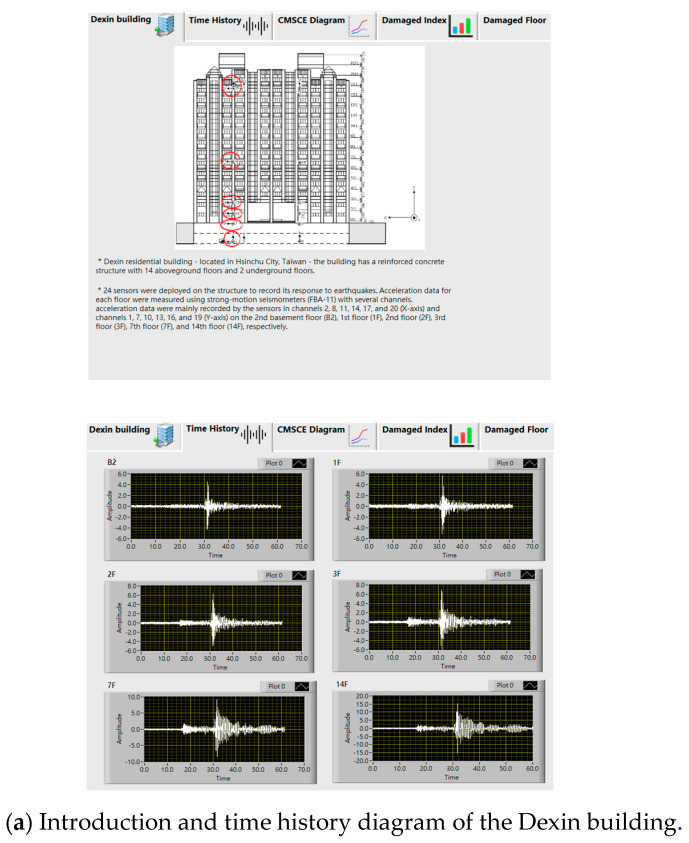

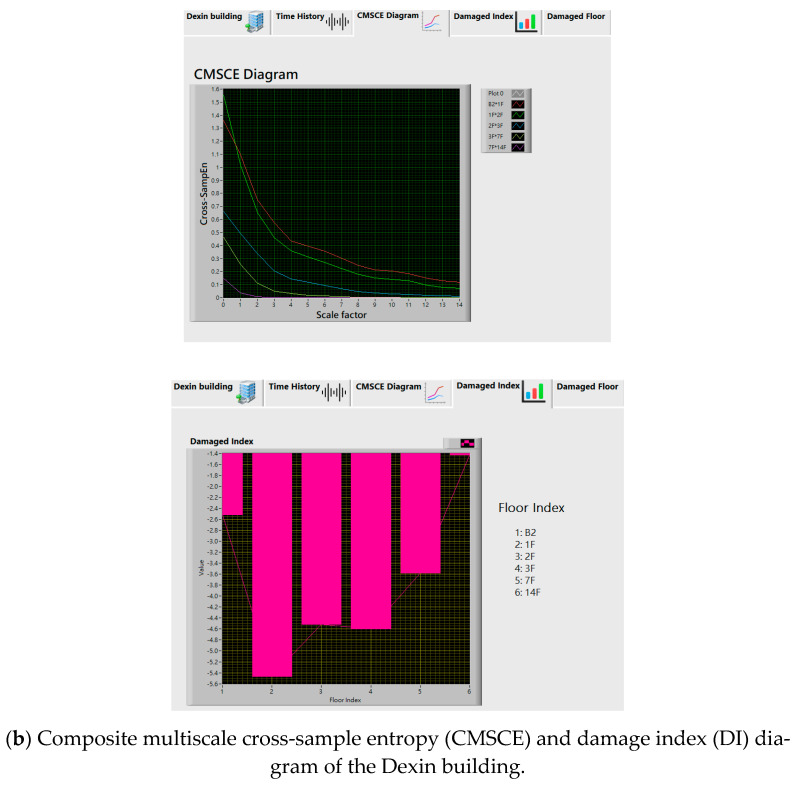
Labview interface of the CMSCE.

A human–machine interface to facilitate on-site processing and provide a real-time visualization of the monitoring result was developed by LabVIEW. The interface integrated the complex operation and visualized the building information, time history, CMSCE curves, and DI graphs, thereby facilitating the task of monitoring the long-term health condition of the building. The operating procedures of LabVIEW for CMSCE analysis are outlined as follows. First, the event data and vibration orientation to be analyzed were selected. Subsequently, the parameters required for CMSCE analysis were entered into the program, including the sample dimension (m), threshold value (r), and sample length. Because CMSCE analysis entails comparing the similarity of two time template spaces, a healthy time template (denoted as a reference) must be determined. The interface displayed a CMSCE diagram that comprised the curves (displayed in distinct colors) of the cross-SampEn between time series generated from two adjacent floors. The curves were plotted as a function of the scale factor (15 scales), thus enabling users to clearly observe the entropy trends for various earthquake events. Bar graphs were also plotted for the DI and visualized on the interface, enabling users to clearly identify the damaged floors. Figure 5 illustrates the curves and graphs visualized by the LabVIEW interface. This interface was divided into four parts, in the order of building introduction, time history diagram, CMSCE curves, and the damage index.

### 3.3. Evaluation of Long-Term Monitoring

#### 3.3.1. CMSCE Diagram Analysis

When the measured peak ground acceleration (PGA) exceeded 4 gal (cm/s^2^), indicating an earthquake event, a trigger mechanism would activate the monitoring system to collect data. Data regarding ambient vibration periods after the earthquake excitation were recorded for CMSCE analysis. Since 1994, data on earthquake-induced accelerations of the Dexin building have been recorded in a database. Earthquakes can be classified into two categories, according to their intensity: minor and major earthquakes. Data regarding minor earthquakes were recorded for long-term structural health monitoring (SHM) from 1994 to 2020 using the proposed system, and the last 12.5 s (2500 points) ambient vibration after an earthquake for each data set was used for CMSCE analysis. An earthquake that occurred on 6 June 1994 was the first event during the 27 year monitoring period. This data set was considered as the reference to represent the healthy condition, and the corresponding structural characteristics were analyzed.

**Figure 6:**
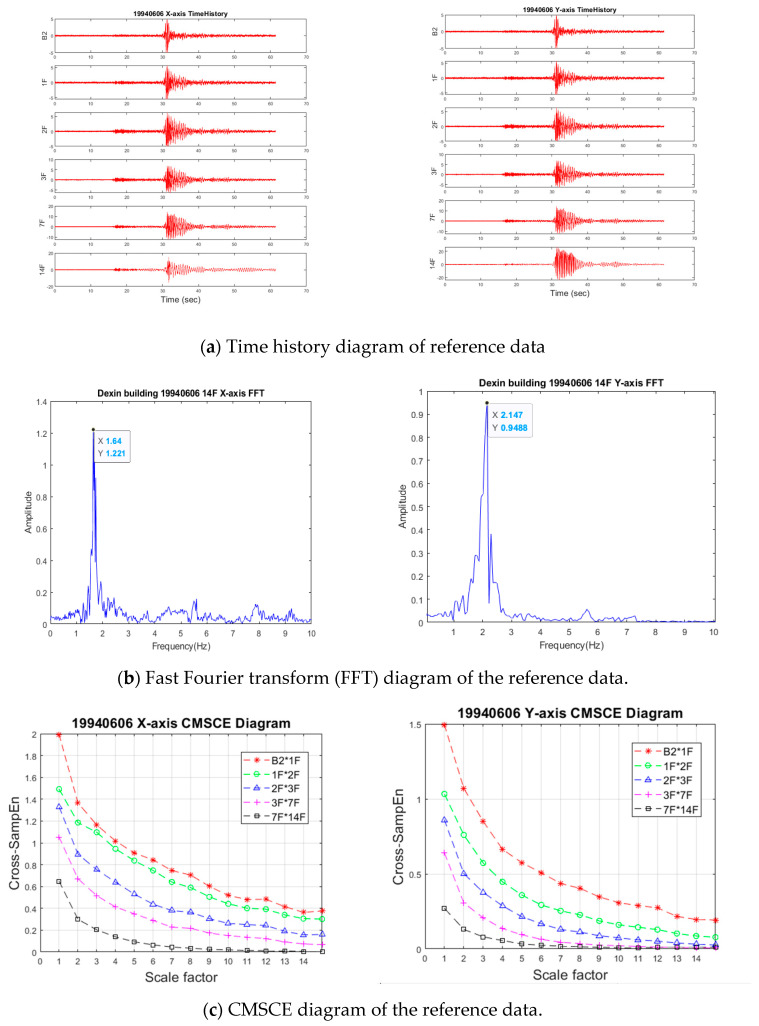
Time history, FFT, and CMSCE diagram of the reference data (6 June 1994).

To analyze the main structural characteristics of the building, including the fundamental frequencies and modes, the fast Fourier transform (FFT) was used to transform time series data from the time domain to the frequency domain. As the fundamental frequency is proportional to $\sqrt{k}$, where *k* represents the global stiffness of the structure, the analysis results were used as the preliminary index to determine whether the structure was damaged due to earthquakes or human use. The FFT results for the reference condition are illustrated in Figure 6b. The main frequencies of the structure were lower than 10 Hz. The first fundamental frequencies in the X- and Y-axes were 1.644 Hz and 2.147 Hz, respectively. These FFT results indicate that the structure was stronger in the Y-axis than it was in the X-axis. According to a previous study on CMSCE, the following parameters were set to analyze the health status of the structure: sample dimension (m) = 4; threshold value (r) = 0.08; and sample length (L) = 2500 points. The analysis results are displayed in Figure 6c. As indicated by the reference CMSCE diagram, the trends of the structural health characteristics were uniformly distributed among the various floors, thereby demonstrating the feasibility of applying the reference data for long-term monitoring.

**Figure 7:**
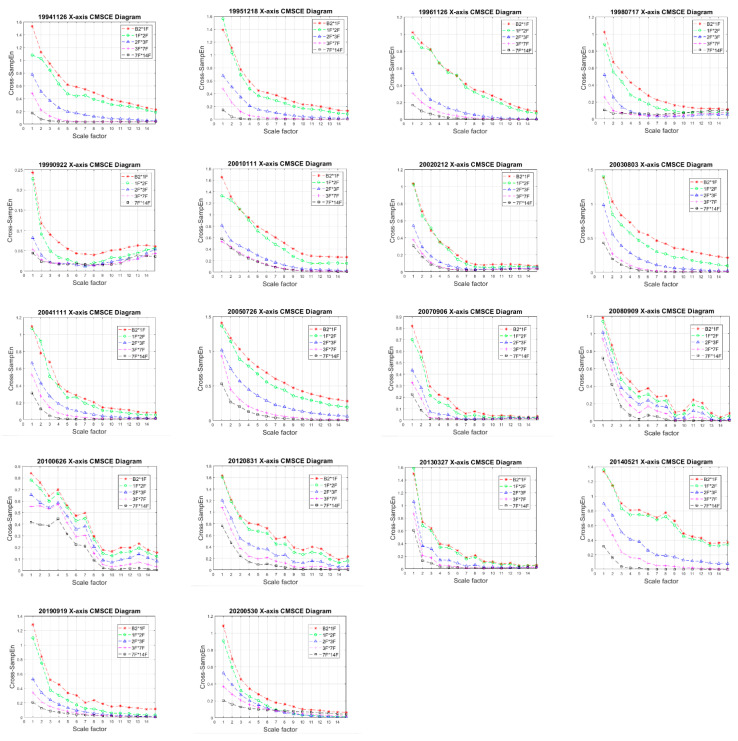
Long-Term CMSCE diagram of Dexin building (X-axis).

**Figure 8:**
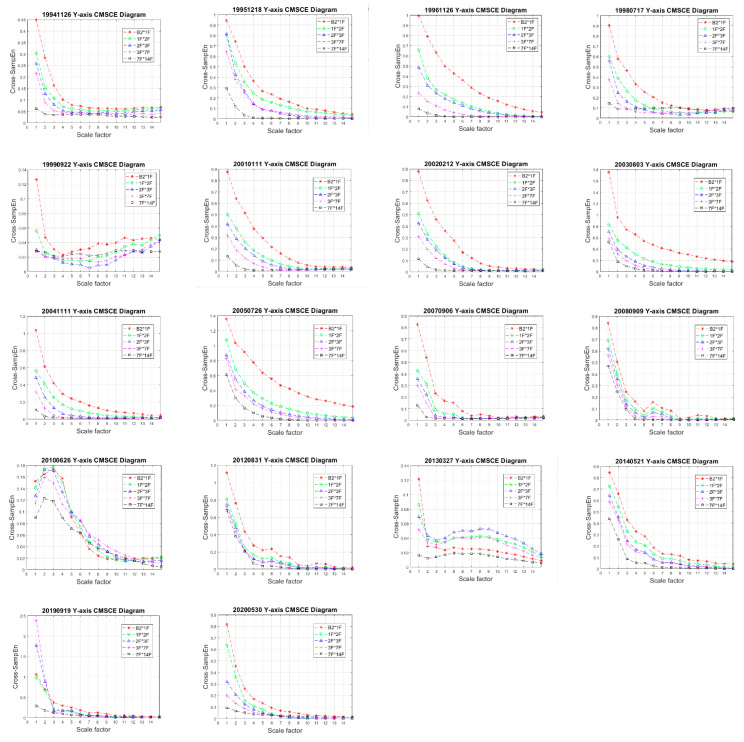
Long-term CMSCE diagram of the Dexin building (Y-axis).

As mentioned, the monitoring database comprised data collected from 1994 to 2020. The health and performance of the structure may have been affected by earthquakes or long-term use. To demonstrate the long-term performance of the CMSCE-based SHM method, this study applied a method to analyze the effect of a minor earthquake event that occurred during this period. Thus, the effects of a total of 18 minor earthquake events were analyzed. The ambient vibration time history measured after earthquake-induced excitation was 12.5 s (2500 points). This structural response was used for CMSCE analysis to evaluate the long-term health condition of the building. Curves of entropy values measured along the X- and Y-axes for the various years are presented in Figure 7 and Figure 8. The X-axis is the scale factor of coarse graining from 1 to 15, and the Y-axis is the cross-SampEn values of each scale factor. Overall, the curves followed similar trends. The entropy values were between 0 and 2; slight differences were observed between values for the various years. The entropy values derived for the lower floors were higher than those derived for the higher floors. For some years, when the scale factor was four, the entropy curves obtained for higher floors did not follow the expected pattern. As the scale factor increased, the entropy values derived for higher floors gradually exceeded those derived for other floors. This phenomenon was also evident in the results obtained for the Y-axis.

#### 3.3.2. DI Analysis

**Figure 9:**
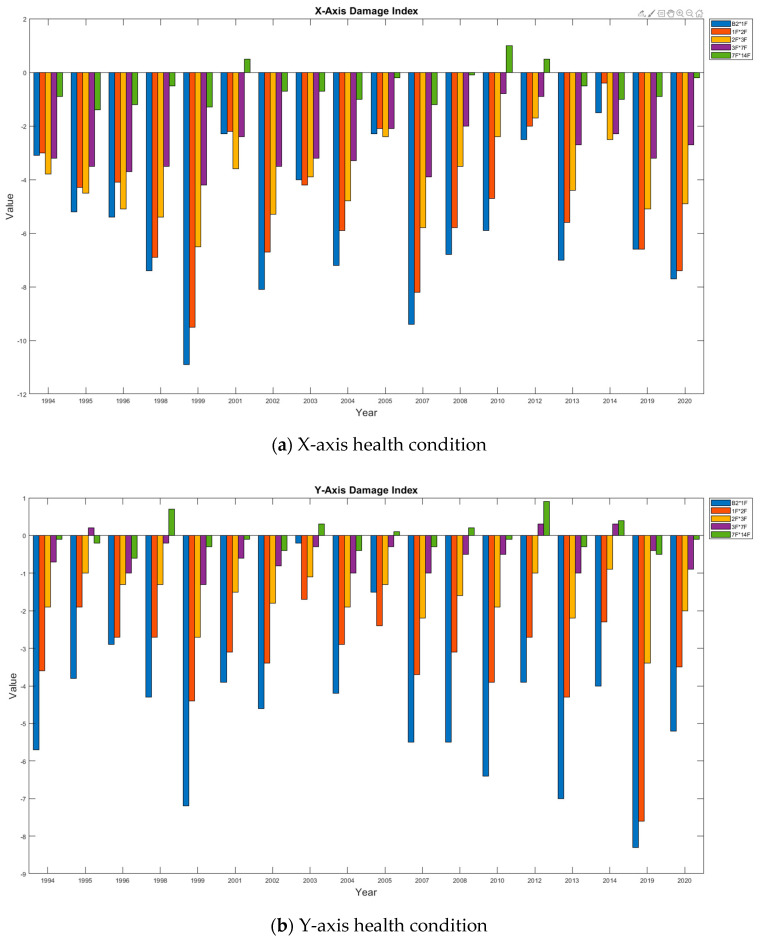
Long-term damage index trend of the Dexin building.

The DI values were calculated for various sections of the building to quantify any possible structural damage. Except for a few marginally positive DI values (<1), the remaining values were negative throughout the 27 year monitoring period. This finding reveals that the building was not damaged. For the cases involving positive DI values, the damage was located on higher floors, such as 3F–7F or 7F–14F. According to the analysis results, data sampled from higher floors were amplified, possibly leading to incorrect diagnoses. Such false alarms can be avoided by adjusting the DI threshold. According to the long-term trends of the CMSCE analysis results, the building was not damaged. Figure 9 displays detailed graphs of the DI values obtained for both the X- and Y-axes.

### 3.4. Earthquake Analysis

Earthquakes have caused severe loss of life and economic losses in the past three decades. Therefore, to diagnose the health condition of the building, this study conducted CMSCE analysis on the acceleration of the structure caused by two major earthquakes that occurred near the monitoring site.

#### 3.4.1. 430 Earthquake

**Figure 10:**
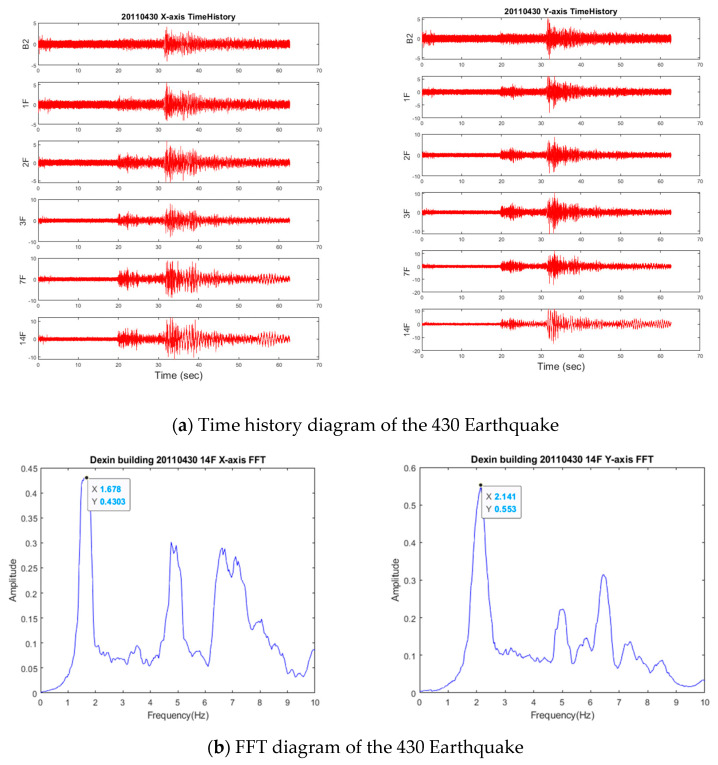

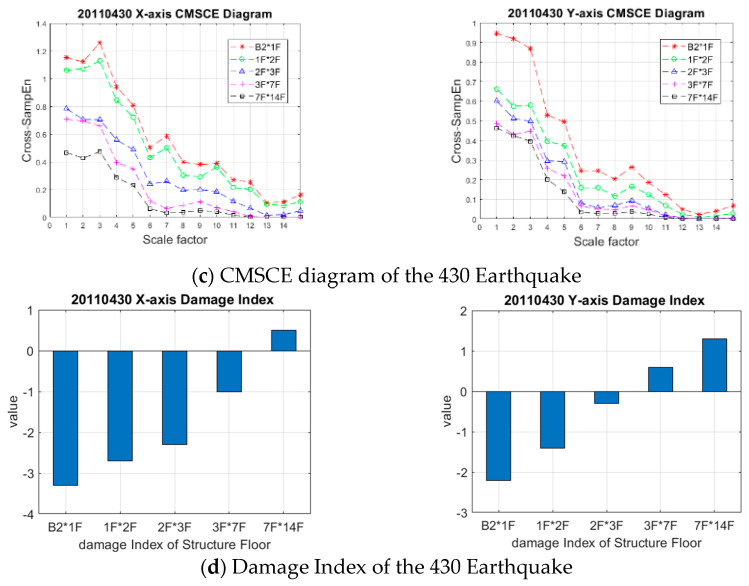
Time history, FFT, CMSCE diagram, and damage index of the 430 Earthquake.

The 430 Earthquake occurred on 30 April 2011. Its epicenter was located at 24.65° N (latitude) and 121.81° E (longitude) in New Taipei City, and its depth of focus was approximately 76 km. The earthquake intensity was recorded to be 5.8 on the Richter scale. As shown in Figure 10a, the PGA values measured from the ground were 5.34 gal and 6.12 gal in the X- and Y-axes, respectively. These values are equivalent to an earthquake of a level 2 intensity. The maximum acceleration measured on 14F was 11.63 in the X-axis and 11.5 in the Y-axis. The vibration period observed for the entire structure was approximately 10 s. FFT analysis revealed that the fundamental vibration frequencies of the building in the two axes after the earthquake were 1.678 Hz and 2.141 Hz (Figure 10b), which were only slightly different from the reference frequencies. Thus, only a marginal difference was observed in the reference frequencies (1.64 Hz and 2.147 Hz). To further evaluate whether the structure was damaged, CMSCE analysis was conducted on ambient vibration data collected after the earthquake event. The CMSCE and DI results are illustrated in Figure 10c,d. In general, the CMSCE curves exhibited only slight fluctuations. Furthermore, the 7F*14F curve revealed a positive DI value of 1.2 in the Y-axis; DI values lower than 1 were ignored, according to the statistics inferred from Figure 9. This finding indicates that the floors between the 7F and 14F may have been slightly damaged during the earthquake and should thus be inspected.

#### 3.4.2. 613 Earthquake

**Figure 11:**
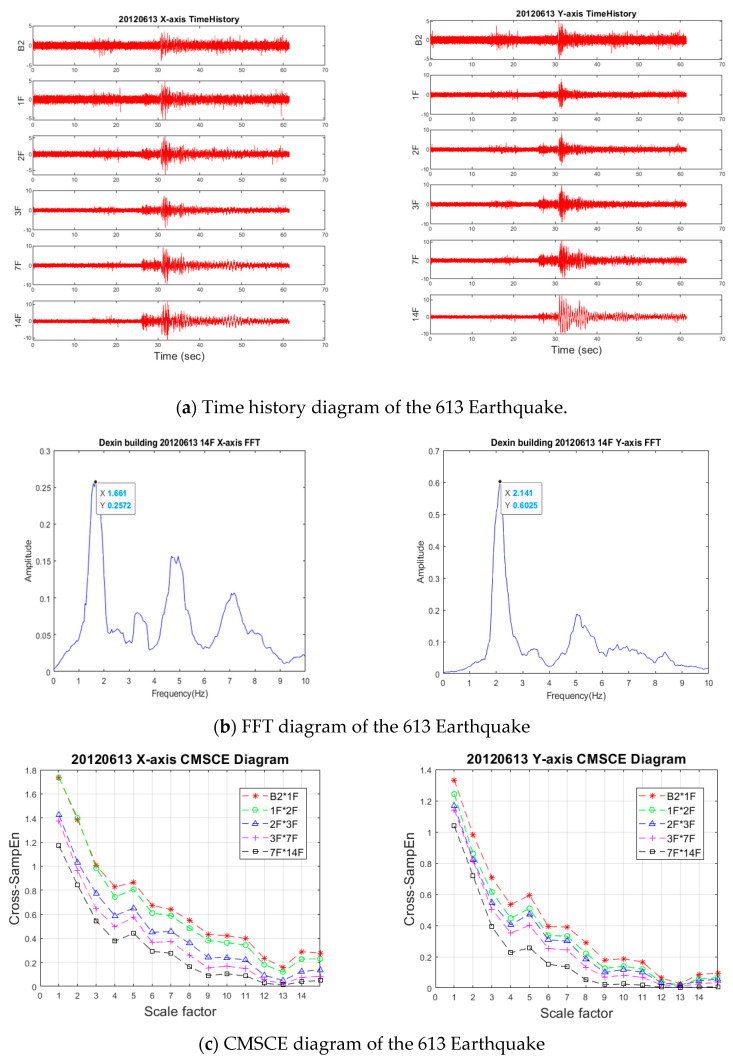

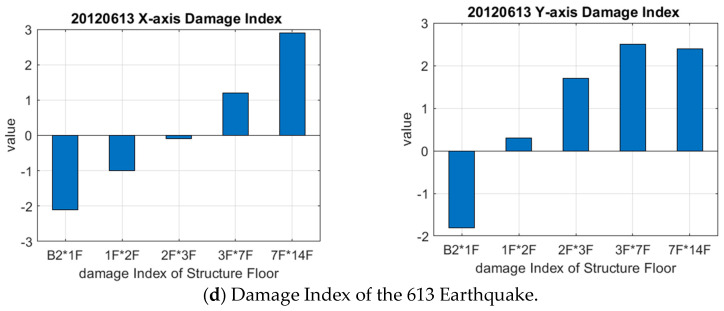
Time history, FFT, CMSCE, and damage index of the 613 Earthquake.

The 613 Earthquake occurred on June 13, 2012 in Jianshi Township, Hsinchu County. The earthquake intensity was recorded to be 4.9 on the Richter scale. The epicenter of the earthquake was at 24.75° N (latitude) and 121.27° E (longitude), with a depth of focus of approximately 6.7 km. This was the first earthquake with a magnitude of more than 4.5 in the area in 58 years. The shallow depth of the earthquake rendered it favorable for SHM evaluation. The time history of the structural vibration is displayed in Figure 11a. The PGA values in the X- and Y-axes were 5.25 gal and 5.34 gal, respectively; these values are equivalent to a level-2 earthquake. Moreover, the maximum acceleration response on the 14F were 12.28 gal and 13.11 gal. The fundamental vibration frequencies of the building in both axes after the earthquake were identified using FFT analysis (Figure 11b). The first fundamental frequencies in the X- and Y-axes were 1.661 Hz and 2.141 Hz, respectively, which were almost the same as the reference frequencies. To detect the damage condition and location, CMSCE analysis was executed using the same settings as those used for the 430 Earthquake. As illustrated in Figure 11c, high entropy values were obtained from the analysis; the maximum entropy values in the X- and Y-axes were approximately 1.8 and 1.4, respectively, which were higher than those observed for the 430 Earthquake. According to the DI values, the conditions of the higher floors (3F*7F and 7F*14F) in the X-axis reached critical levels. The 2F*3F, 3F*7F, and 7F*14F curves also revealed DI values higher than 1. These values indicate that the health condition of the higher floors of the residential building should be examined.

## 4. Conclusions

Entropy analysis has been extensively used in various fields. In this study, a CMSCE system was applied to analyze the long-term structural health of a residential building over a period of 27 years. CMSCE, which improves the reliability of CMSE, was adopted as the core algorithm for SHM diagnosis software, implemented using LabVIEW. The effectiveness of the system for long-term monitoring and earthquake analysis was verified. A data set collected in 1994 was selected as the reference for the executed CMSCE analysis. Entropy curves were plotted for 18 collected minor earthquake events, and the corresponding DI values were plotted in graphs to demonstrate the long-term health condition of the building. The DI graphs exhibited a stable trend, indicating the health condition of the building. Moreover, two large earthquake events that occurred near the monitoring site were analyzed. The results revealed that the entropy values increased slightly after the major earthquakes. Positive DI values were derived for higher floors. These values could provide an early and efficient warning of structural instability. The CMSCE-based SHM system demonstrated high stability and practicability over a long monitoring period.

## Figures and Tables

**Table 1:** The numbering of channels on each floor.

Channel	Axis	Location
CH01	Y	B2 left floor (near elevator)
CH02	X	B2 left floor (near elevator)
CH03	Z	B2 left floor (near elevator)
CH04	Z	B2 left floor (near pipeline)
CH05	Y	B2 right floor
CH06	Z	B2 right floor
CH07	Y	1F left floor (near elevator)
CH08	X	1F left floor (near elevator)
CH09	Y	1F floor
CH10	Y	2F left floor
CH11	X	2F left floor
CH12	Y	2F right floor
CH13	Y	3F left floor
CH14	X	3F left floor
CH15	Y	3F right floor
CH16	Y	7F left floor
CH17	X	7F left floor
CH18	Y	7F left floor
CH19	Y	14F left floor
CH20	X	14F left floor
CH21	Z	14F left floor
CH22	Z	14F left floor
CH23	Y	14F left floor
CH24	Z	14F left floor
